# Identification of a novel splice mutation in CTNNB1 gene in a Chinese family with both severe intellectual disability and serious visual defects

**DOI:** 10.1007/s10072-019-03823-5

**Published:** 2019-03-30

**Authors:** Hui Wang, Yiqi Zhao, Liwei Yang, Shuai Han, Ming Qi

**Affiliations:** 1 Department of Obstetrics, Zhejiang Provincial People’s Hospital, People’s Hospital of Hangzhou Medical College, No. 158, Shangtang Road, Hangzhou, Zhejiang Province China; 2 Department of Cell Biology and Medical Genetics, School of Medicine, Zhejiang University, Hangzhou, China; 3 Assisted Reproduction Unit, Department of Obstetrics and Gynecology, Sir Run Run Shaw Hospital, School of Medicine, Key Laboratory of Reproductive Dysfunction Management of Zhejiang Province, Zhejiang University, Hangzhou, China; 4 Department of Pathology and Laboratory Medicine, University of Rochester Medical Center, Rochester, NY USA

**Keywords:** *CTNNB1* gene, Intellectual disability, Visual defects, Splice mutation

## Abstract

The *CTNNB1* gene encode the β-catenin protein which is a core unit of the cadherin/catenin multiprotein complex. The loss-of-function mutation of the *CTNNB1* gene recently has been confirmed as a cause of intellectual disability. Previous studies have found that patients with *CTNNB1* gene mutation may have other clinical manifestation such as microcephaly, abnormal facial features, motor and language delays, and mild visual defects. Here, we reported a 27-year-old Chinese pregnant woman with a severe intellectual disability and serious visual defects who was detected with a novel splice mutation (c.734+1G>A) in *CTNNB1* gene by whole-exome sequencing and confirmed by Sanger sequencing. Further investigation showed that the variant was inherited from her mother with similar phenotypes. This report not only helps to expand the mutant spectrum of the *CTNNB1* gene but also prompts a new insight into genetic diagnosis in patients with both serious intellectual disability and visual defects.

**Electronic supplementary material:**

The online version of this article (10.1007/s10072-019-03823-5) contains supplementary material, which is available to authorized users.

## Introduction

The *CTNNB1* gene encodes the β-catenin protein which is a sub-unit of the cadherin/catenin multiprotein complex. Previous studies find *CTNNB1* mutation related to several cancers [1]. Recently, a large-scale sequencing firstly identified *CTNNB1* loss-of-function mutations as the cause of intellectual disability (ID) [2]. Other studies report different mutations in patients which phenotypes include ID, craniofacial anomalies, speech delay microcephaly, and mild visual disturbances like strabismus and hyperopia [3–5]. Our study describes a novel mutation of the *CTNNB1* gene in a family who presented with serious ID and visual disturbances.

## Case report

**Fig. 1:**
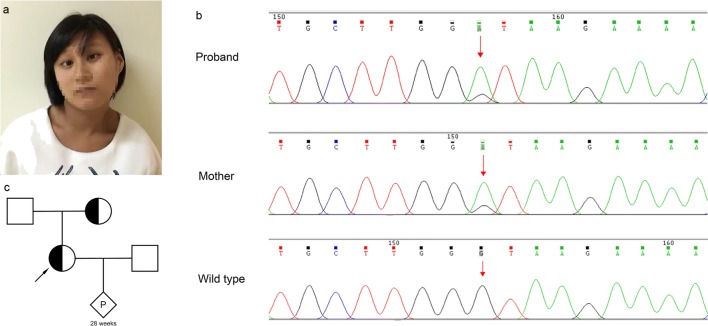
Photographs of the proband, mutations in the *CTNNB1* gene, and the pedigrees of the families. **a** The proband had mildly abnormal craniofacial features with face involuntary movements, severe esotropia, rotation of the head, and significant motor delay and language retardation. **b** Sanger sequence showed a heterozygous splice mutation (c.734+1G>A) in the proband and her mother. Red arrows, mutant bases. **c** The pedigrees of the families

The proband was a 27-years-old Chinese pregnant woman who attended to our department at 20th gestational week for prenatal diagnosis because of the possible maternal ID. She also had severe esotropia, involuntary rotation of the head, and significant motor delay and language retardation (Fig. 1a), which appeared and got worsen since her birth. Neurologist and ophthalmologist were invited for a professional examination.

The neurologic examination found she had peripheral hypertonia with deep tendon hyperreflexia and Babinski sign positive, ataxic gait, and paroxysmal dystonic of head and neck, especially right-side rotation of head (Online Resource 1). Ceruloplasmin test, immunologic tests, brain MRI, and routine laboratory tests revealed nothing remarkable. Electroencephalograph (EEG) was mildly abnormal without typical epileptiform discharge and electromyography (EMG) was normal. Wechsler intelligence test showed severe ID with IQ score 28. The ophthalmologist found the woman had serious esotropia of both eyes, no reaction to light, and a complete lack of vision accompanied with nystagmus, lens, and vitreous opacities on oculus sinister. Complete retinal detachment and eyeball atrophy on her left eye were confirmed by fundus ultrasound.


Online Resource 1(MP4 9892 kb)


For further investigation, whole-genome low-coverage sequencing (WGLCS) and whole-exome sequencing (WES) were performed to detect the potential pathogenic factors besides Karyotype (Online Resource 2). A novel mutation (c.734+1G>A) in the *CTNNB1* gene was detected by WES and confirmed by Sanger sequencing (Fig. 1b).

**Fig. 2:**
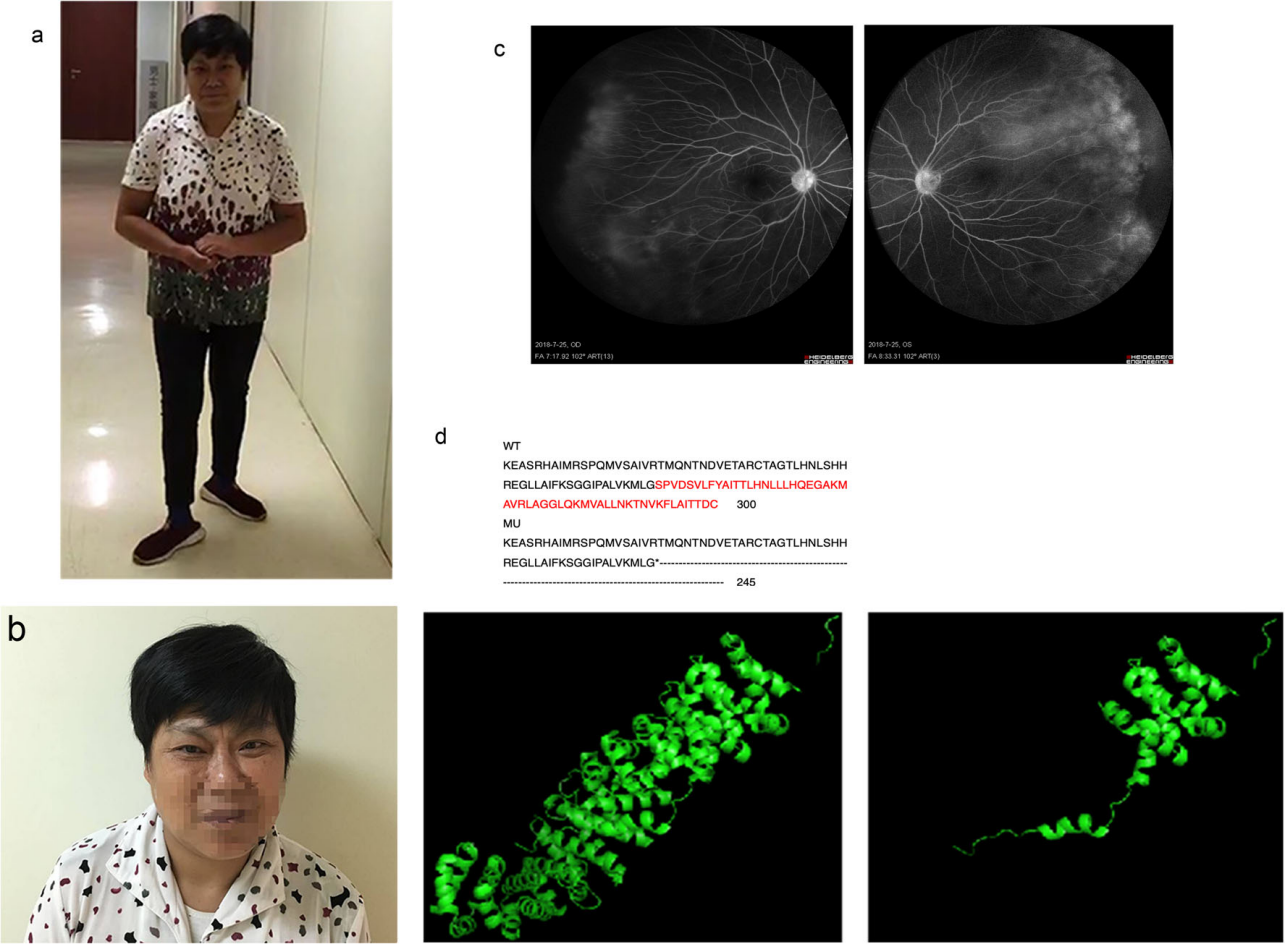
Photographs of proband’s mother and her fundus fluorescein angiography, expected effects on amino acid sequences, and protein functionality. **a** and **b** The proband’s mother had similar but slighter abnormal craniofacial features like a grimace, esotropia, rotation of the head, and ataxic gait. **c** Fundus fluorescein angiography showed exudative vitreoretinopathy on the temporal side in both eyes of proband’s mother. Red boxes, exudative vitreoretinopathy. **d** Amino acid sequences of the wild-type (WT) and mutation (MU) showed the mutation leading to truncation of amino acid by Clustal Omega. Three-dimensional modeling of the normal CTNNB1 protein (left) and mutant CTNNB1 protein (right) using PyMol software


To our interest, we learned that the proband’s mother has similar but slighter clinical manifestations (Fig. 1c; Online Resource 3). She was invited to our hospital for detailed examinations. She was 49 years old with ID (IQ score 40) and had similar neurological clinical manifestations (Fig. 2a, b) and unremarkable brain MRI and EEG. Ophthalmologic examination indicated impaired visual acuity (logarithmic visual acuity chart 0.08 in OD and 0.1 in OS) and esotropia. Fundus fluorescein angiography was then performed and displayed the exudative vitreoretinopathy (EVR) on the temporal side in both of her eyes (Fig. 2c). Sanger sequencing was carried out and confirmed the same splice mutation (c.734+1G>A) in the *CTNNB1* gene in the proband’ s mother (Fig. 1c).

Online Resource 3(MP4 13,289 kb)


The splice mutation (c.734+1G>A) on CTNNB 1 gene has not been reported by any research or recorded by any related gene mutation database, such as HGMD, Clinvar, and LOVD and leads to a truncated CTNNB1 protein of 245 in comparison with the wide-type sequence of 781 amino acids. We performed three-dimensional modeling change of the truncated CTNNB1 protein (Fig. 2d), which loses the very important functional domain interactive with BCL9 (B cell CLL/lymphoma 9) and SCRIB indicated by Unipro database. As BCL9 and SCRIB both participate in the Wnt signaling pathway, so we infer that the splice mutation (c.734+1G>A) affect the structure and function of a β-catenin protein which may influence the Wnt signaling pathway and causing a series of clinical manifestations. So, our further research should elucidate how the mutation affects the function of β-catenin and the precise pathogenic molecular mechanisms.

## Discussion

The *CTNNB1* gene encodes a β-catenin protein which is a central component of the cadherin/catenin complex. β-catenin protein participates in cell adhesion and nuclear signaling which mainly involves in the Wnt protein-mediated signal pathway. Previous studies declare *CTNNB1* mutation is related to several cancers such as hepatocellular carcinoma, medulloblastoma, ovarian cancer, and pilomatricoma [1]. It was firstly connected to ID since 2012 by De Ligt J [2] and then, a series of studies report more than 20 loss-function-mutations of the CTNNB1 gene which may lead to ID [6]. As known, human β-catenin contains 781 amino acids and is subdivided into three domains: an amino-terminal domain (NTD), a central region containing 12 armadillo (ARM) repeats (residues 138–664), and a carboxy-terminal domain (CTD). However, by which mechanism, the truncated protein identified by this study may influence the Wnt signaling; it is worth further research.

Phenotypic characteristics of *CTNNB1* mutation patients include ID, postnatal microcephaly, mild craniofacial particularities, and neurological disorders such as peripheral hypertonia, motor, and language development delay (Online Mendelian Inheritance in Man, OMIM# 615075). Mild visual disturbances were also reported such as astigmatism, hyperopia, and strabismus. Our case also indicated similar features but the proband suffers from complete retinal detachment and her mother also has severely impaired visual acuity. Panagiotou et al. found the *CTNNB1* gene can result in exudative vitreoretinopathy with no neurological disorders [7]. However, one 3-year-old Chinese boy initially presented with EVR showed facial dysmorphism and global developmental delay during follow-up and WES identified a de novo 1-bp insertion (c.1434_1435insC, Glu479ArgfsTer18) in *CTNNB1* gene (OMIM *116806.0023). Niu Li et al. also reported a 15-month-old Chinese boy with retinal detachment and development delay, who was negative for mutation related to the exudative vitreoretinopathy but a heterozygous nonsense mutation (c.1627C>T, p. Gln558X) in *CTNNB1* gene was found [6]. Combined with our case, we suppose that *CTNNB1* gene mutation not only can cause ID or EVR singly but also may synchronously result in these series phenotypes.

In conclusion, our study identifies a novel heterozygous splice mutation in the *CTNNB1* gene (c.734+1G>A) in a Chinese family with both severe ID and visual disturbances. It helps to expand the mutant spectrum of the *CTNNB1* gene and confirms that different types of mutation in *CTNNB1* may be associated with ID and visual disturbances.

## Electronic Supplementary Material

Online Resource 2 (PDF 263 kb)
