# Supplementary material for: Identification of a novel splice mutation in CTNNB1 gene in a Chinese family with both severe intellectual disability and serious visual defects
Source: Neurol Sci. 2019 Mar 30;40(8):1701–4. doi: 10.1007/s10072-019-03823-5 (PMC6647480; doi:10.1007/s10072-019-03823-5)
Supplement: Supplementary file 3 — (PDF 263 kb) [file 10072_2019_3823_MOESM2_ESM.pdf]

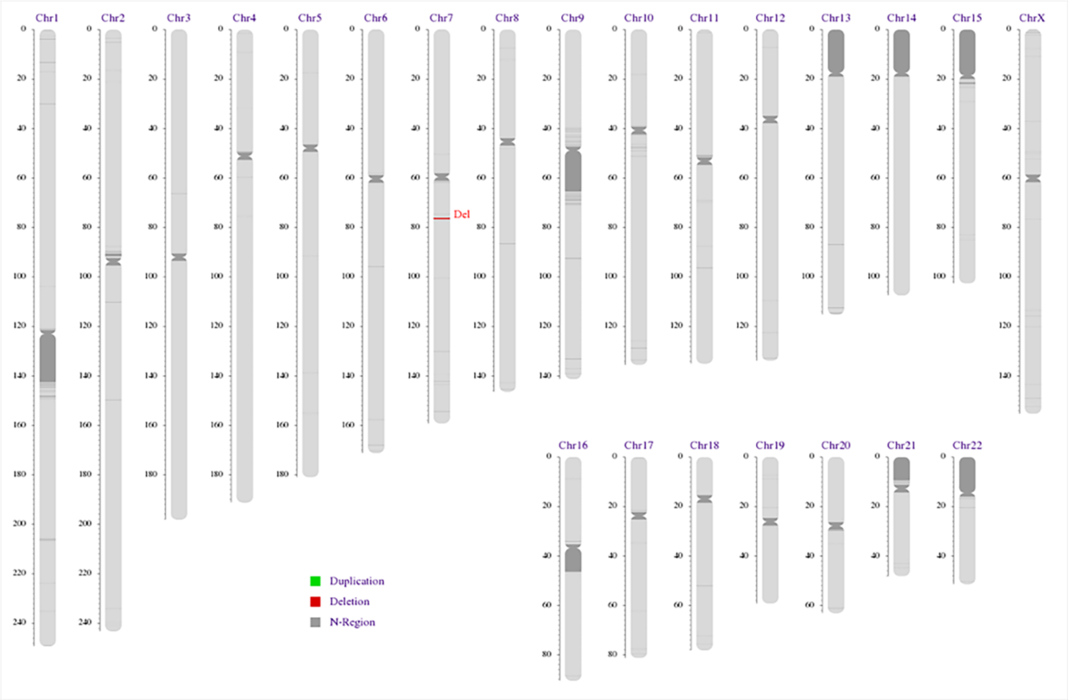

Fig.1 Whole genome low coverage sequencing found a 469.94kb deletion with undetermined significance

WGLCS finds a 469.94 Kb deletion located at 7q11.23(chr7:76,123,202-7 6,593,144) in the proband which didn’t contain any reported pathogenic gene. Further investigation shows the deletion only existed in the proband but not in her mother and grandmother.

1. Pober BR(2010) Williams–Beuren Syndrome. N Engl J Med 362:239-252. https://doi.org/10.1056/NEJMra0903074

2. Ramocki MB, Bartnik M, Szafranski P, Kolodziejska KE, Xia Z, Bravo J, Miller GS, Rodriguez DL, Williams CA, Bader PI, Szczepanik E, Mazurczak T, and 16 others(2010)Recurrent distal 7q11.23 deletion including HIP1 and YWHAG identified in patients with intellectual disabilities, epilepsy, and neurobehavioral problems. Am. J. Hum. Genet. 87: 857-865. https://doi.org/10.1016/j.ajhg.2010.10.019
